# Supplementary material for: HISTONCHO: A dataset of intervention histories for onchocerciasis control & elimination in sub-Saharan Africa
Source: Sci Data. 2026 Feb 19;13:480. doi: 10.1038/s41597-026-06852-w (PMC13031844; doi:10.1038/s41597-026-06852-w)
Supplement: Supplementary file 1 — Supplementary information file [file 41597_2026_6852_MOESM1_ESM.pdf]

Supplementary Information File 1

Title

HISTONCHO: A dataset of intervention histories for onchocerciasis control & elimination in sub-Saharan Africa

Authors

Matthew A. Dixon[1*], Martin Walker[1,2], Aditya Ramani[2], Jenna E. Coalson[3], Emily Griswold[3], Gregory S. Noland[3], Andrew Tate[4], Emeka Makata[5], Ahmed M. A. Ali[6], Jorge Cano[7], Paul Bessell[8], Claudio Fronterrè[9], Raiha Browning[10], Wilma A. Stolk[11] & Maria-Gloria Basáñez[1*]

Affiliations

[1]MRC Centre for Global Infectious Disease Analysis, and London Centre for Neglected Tropical Disease Research, Department of Infectious Disease Epidemiology, School of Public Health, Imperial College London, 90 Wood Lane, London, W12 0BZ, UK

[2]Department of Pathobiology and Population Sciences, Royal Veterinary College, Hawkshead Lane, Hatfield, Hertfordshire, AL9 7TA, UK

[3]The Carter Center, One Copenhill, 453 John Lewis Freedom Parkway NE, Atlanta, GA 30307-1406, USA

[4]Sightsavers, 35 Perrymount Road, Haywards Heath, RH16 3BW, UK

[5]Federal Ministry of Health & Social Welfare, Federal Republic of Nigeria, Federal Secretariat Complex, Phase III, Shehu Shagari Way, Central Business District, FCT Abuja, Nigeria

[6] Republic of the Sudan Federal Ministry of Health, P.O. Box 303 Khartoum, Sudan

[7]Expanded Special Project for Elimination of Neglected Tropical Diseases (ESPEN), World Health Organization Regional Office for Africa (AFRO), Brazzaville, Republic of Congo

[8]Epi Interventions Ltd, White Lodge Pencaitland, Tranent, EH34 5BQ, UK

[9]Centre for Health Informatics, Computing and Statistics (CHICAS), Lancaster University, Lancaster, LA1 4YW, UK

[10]Department of Statistics, University of Warwick, Coventry, CV4 7AL, UK

[11]Department of Public Health, Erasmus MC, University Medical Center Rotterdam, Rotterdam, The Netherlands.

*e-mail: m.dixon15@imperial.ac.uk (MAD); m.basanez@imperial.ac.uk (MGB).

Table S1. HISTONCHO database variable names, descriptions and sources of information for the 34 variables included

Variable name	Description	Reference; Main text Table(s) & Figure(s)
IU_ID_MAPPING	ESPEN IU ID, updated to account for changes in Implementation Unit demarcations	ESPEN (2022)[1]
IUs_NAME_MAPPING	Name of Implementation Unit, updated to account for changes in Implementation Unit demarcations	ESPEN (2022)[1]
IU_CODE_MAPPING	ESPEN IU alphanumeric code (ISO3Code+ADMIN1+IU_ID), updated to account for changes in Implementation Unit demarcations	ESPEN (2022)[1]
ADMIN0ISO3	Country ISO3 Code	ESPEN (2022)[1]
Endemicity	Reported Onchocerciasis Endemicity status through Joint Reporting Form (JRF) after final correction	ESPEN (2022)[1]
MAX_Endemicity	Defines the 'maximum' level of classification in the "Endemicity" for an IU across all ESPEN years (2013-2022) = i) 'Endemic' if this designation appears between 2013-2022, ii) 'Unknown', iii) 'Not reported', iv) 'Non-endemic'	–
Control_Prog	Indicates whether the IU is considered under the former Onchocerciasis Control Programme in West Africa or African Programme for Onchocerciasis Control = i) 'OCP', ii) 'APOC'	Table 1, Table 2 & Figure 1
OCP_PHASE	Defines which OCP phase an IU belongs to (only former OCP countries) = i) 'Former OCP Phase I - 1975', ii) 'Former OCP Phase II - 1976', iii) 'Former OCP Phase III East - 1977', iv) 'Former OCP Phase III West - 1977', v) 'Former OCP Western Extension - 1989', vi) 'Former OCP Western Extension - 1990', vii) 'Former OCP Western Extension - 1990 (March)', viii) 'Former OCP Southern Extension - 1988', ix) 'Former OCP Southern Forest Extension-1990', x) 'Former OCP: Non-Control'	O'Hanlon *et al.*[2]; Figure 1
SIZ_Label	Indicates whether the IU is within a former Special Intervention Zone (SIZ) for that year (only former OCP countries) = i) 'SIZ', ii) 'NA' (non-SIZ)	Table 1 & Figure 1
Endemicity_Baseline	Onchocerciasis endemicity classification = i) 'Hypoendemic', ii) 'Mesoendemic', iii) 'Hyperendemic' based on the mean (point estimate) pre-control microfilarial prevalence for OCP countries or converted from Rapid Epidemiological Mapping of Onchocerciasis (REMO) nodule prevalence for APOC countries	O'Hanlon *et al.*[2]; Zouré *et al.*[3]; Coffeng *et al.*[4]; Coffeng[5]
Year	Year	ESPEN (2022)[1]
PopTot	Reported/estimated total population for 2013-2022	ESPEN (2022)[1]
PopPreSAC	Reported/estimated pre-school-age children population for 2013-2022	ESPEN (2022)[1]
PopSAC	Reported/estimated school-age children population for 2013-2022	ESPEN (2022)[1]
PopAdult	Reported/estimated adult population for 2013-2022	ESPEN (2022)[1]
PopReq	Reported total population requiring treatment (JRF) for 2013-2022. JRF: Joint Reporting Form	ESPEN (2022)[1]
PopTrg	Total population targeted for treatment for 2013-2022	ESPEN (2022)[1]
PopTreat	Total population treated for 2013-2022	ESPEN (2022)[1]
MDA_Scheme	Mass drug administration (MDA) scheme implemented (which provides maximum coverage across different schemes) including values for ivermectin (IVM) = i) 'IVM'; ii) albendazole-ivermectin	ESPEN (2022)[1] (for 2013–2022); APOC (2015)[6] (for 1975-

	= 'ALB-IVM'; iii) 'Not delivered'; iv) 'Pre-ESPEN IVM' for 1975-2012'; v) Not applicable ='NA'*	2012); Table 1 & Table 2
Cum_MDA	Cumulative MDA rounds since the inception of MDA interventions with ivermectin	ESPEN (2022)[1]
Cov	Programme coverage for total population (total treated/total pop targeted for treatment) 2013-2022, expressed as a proportion	ESPEN (2022)[1]
EpiCov	Epidemiological coverage for total population (total treated/total pop requiring treatment) for years 2013-2022, expressed as a proportion	ESPEN (2022)[1]
Vector_Control	Indicates the presence of vector control = i) '1' (present), ii) '2' (vector elimination or disappearance), iii) '0' (no vector control)	Table 1 & Table 2
Non-CDTI_MDA	Indicates annual MDA before Community-Directed Treatment with Ivermectin (CDTI) = i) '1' (annual MDA), ii) '0' (no MDA)	Table 1 & Table 2
CDTI_MDA	Indicates MDA once CDTI commences = i) '1' (CDTI MDA), ii) '0' (no CDTI MDA)	APOC (2015)[6]; Table 1 & Table 2
Biannual_CDTI_MDA	Indicates biannual CDTI MDA = i) '1' (biannual MDA), ii) '0' (no biannual MDA)	Table 1 & Table 2
Number_Rounds	Indicates the number of yearly MDA rounds = i) '1', ii) '2' depending on frequency (sum of "Non-CDTI_MDA", "CDTI_MDA" and "Biannual_CDTI_MDA")	–
Cov_Raw	Raw coverage values (continuous numerical variable), expressed as a proportion	ESPEN (2022)[1]; APOC (2015)[6]; Table 2
Cov_Source	Indicates the source of the coverage data informing "Cov_Raw"	ESPEN (2022)[1]; APOC (2015)[6]; Table 2
Cov_Cat	Categorised coverage value informed by "EpiCov" and/or "Cov_Raw" = i) '0.52' for "Non-CDTI_MDA" = '1'; ii) '0.25' for "CDTI_MDA" = '1' when "Epi_Cov" and/or "Cov_Raw" is >0 but <0.65, iii) '0.65' for "CDTI-MDA" =1 when "Epi_Cov" and/or "Cov_Raw" ≥0.65	–
MDA_VC_Mapping	Summarises the type of intervention history for the IU (across all years) = i) 'Annual MDA' where only annual ivermectin MDA has been implemented; ii) 'Biannual MDA' if there has been any biannual MDA, iii) 'Annual MDA and vector control', iv) 'Biannual MDA and vector control', v) 'Treatment-naïve' (IUs without any intervention)	Figure 5
Cum_MDA_Rev	Revised cumulative number of MDA rounds based on back-dated histories (calculated using "Number_rounds")	Figure 6
Co_endemicity	Indicates co-endemicity status with loiasis and/or lymphatic filariasis (LF) = i) 'Only Oncho', ii) 'Oncho and LF', iii) 'Oncho and Loa hypo' (hypoendemic loiasis), iv) 'Oncho and Loa meso' (mesoendemic loiasis), v) 'Oncho and Loa hyper' (hyperendemic loiasis), vi) 'Oncho and LF and Loa hypo', vii) 'Oncho and LF and Loa meso', viii) 'Oncho and LF and Loa hyper'. (For Oncho pre-control endemicity see "Endemicity_Baseline")	Figure 7
Trt_Status_2022	Indicates status of the IU in 2022 = i) 'MDA continues', ii) 'MDA stopped', iii) 'some previous IVM; potentially requiring OEM and/or SM', iv) 'some previous IVM; potentially requiring OEM and/or SM*', v) 'Treatment-naïve; potentially requiring onchocerciasis elimination mapping (OEM) and/or suitability mapping (SM)' (*) indicates in former OCP areas	Figure 2 & Figure 8

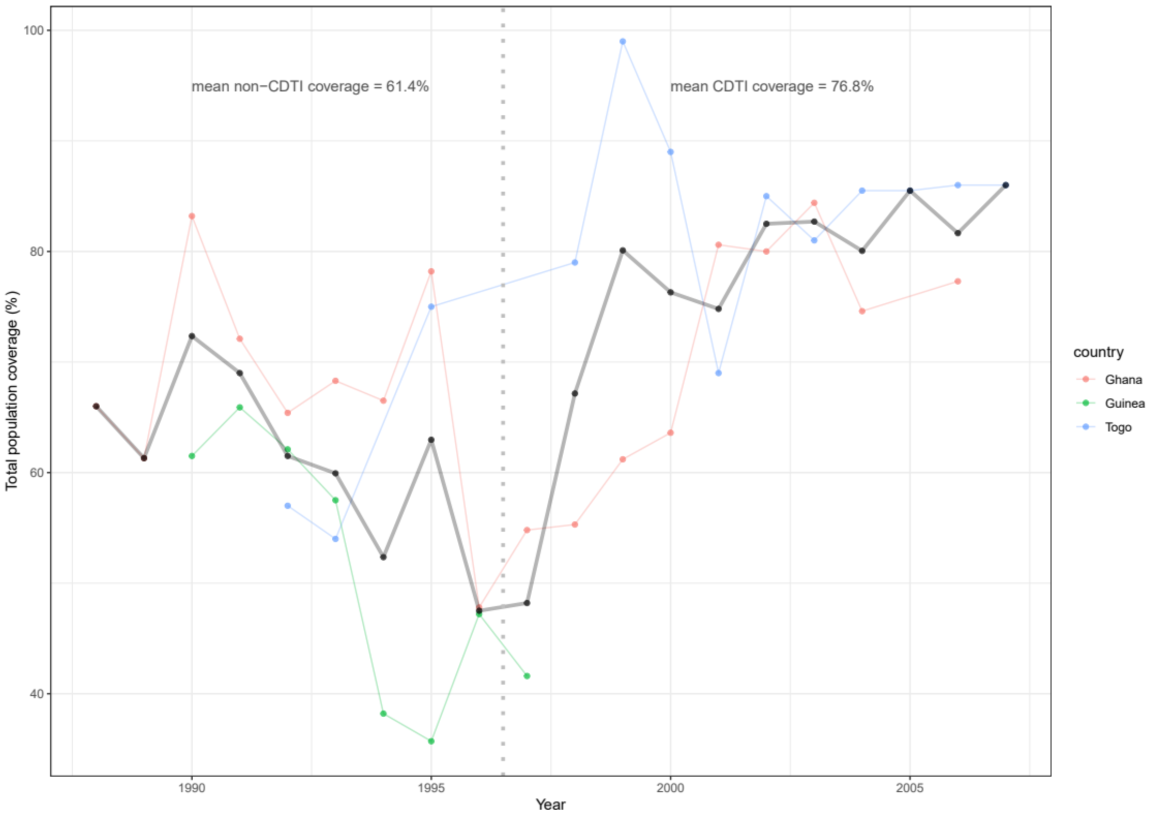

Fig. S1 Coverage temporal trends for three indicator villages within Special Intervention Zones (SIZ) of the former Onchocerciasis Control Programme in West Africa (OCP). The villages are: Asubende (Ghana, red), Serekoroba (Guinea, green) and Titira (Togo, blue). Coverage of total population is plotted for each year during the period 1988–1996, prior to the adoption of Community-Directed Treatment with Ivermectin (non-CDTI) and during 1997–2007, subsequent to CDTI implementation. The thick grey line is the mean coverage for each year. The dashed vertical light grey line signals the switch in ivermectin distribution from non-CDTI to CDTI. On average, during the non-CDTI period the coverage was 61.4% in contrast to the average coverage of 76.8% for the CDTI period, suggesting that CDTI led to an increase in coverage of approximately 25% in these examples[7].

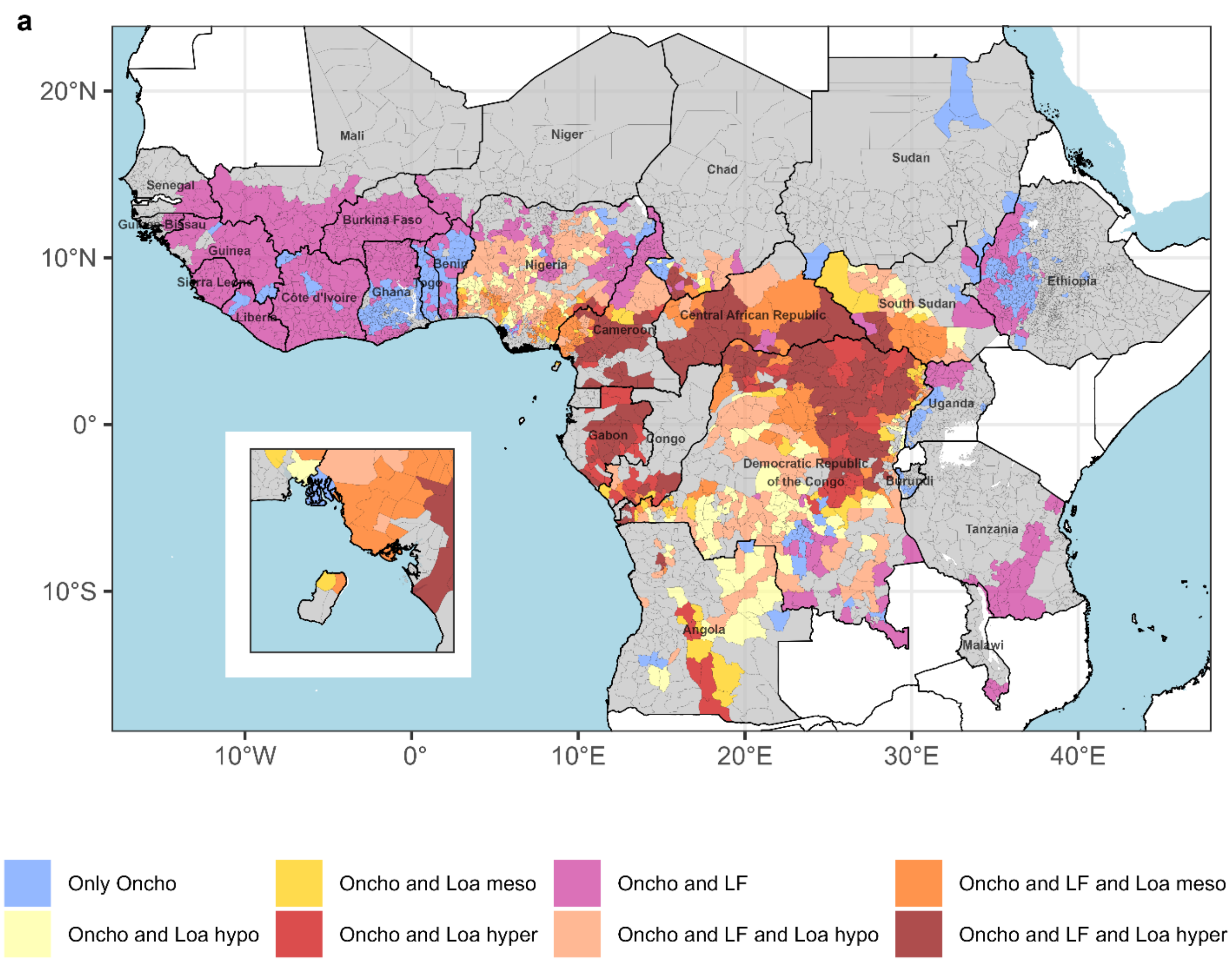

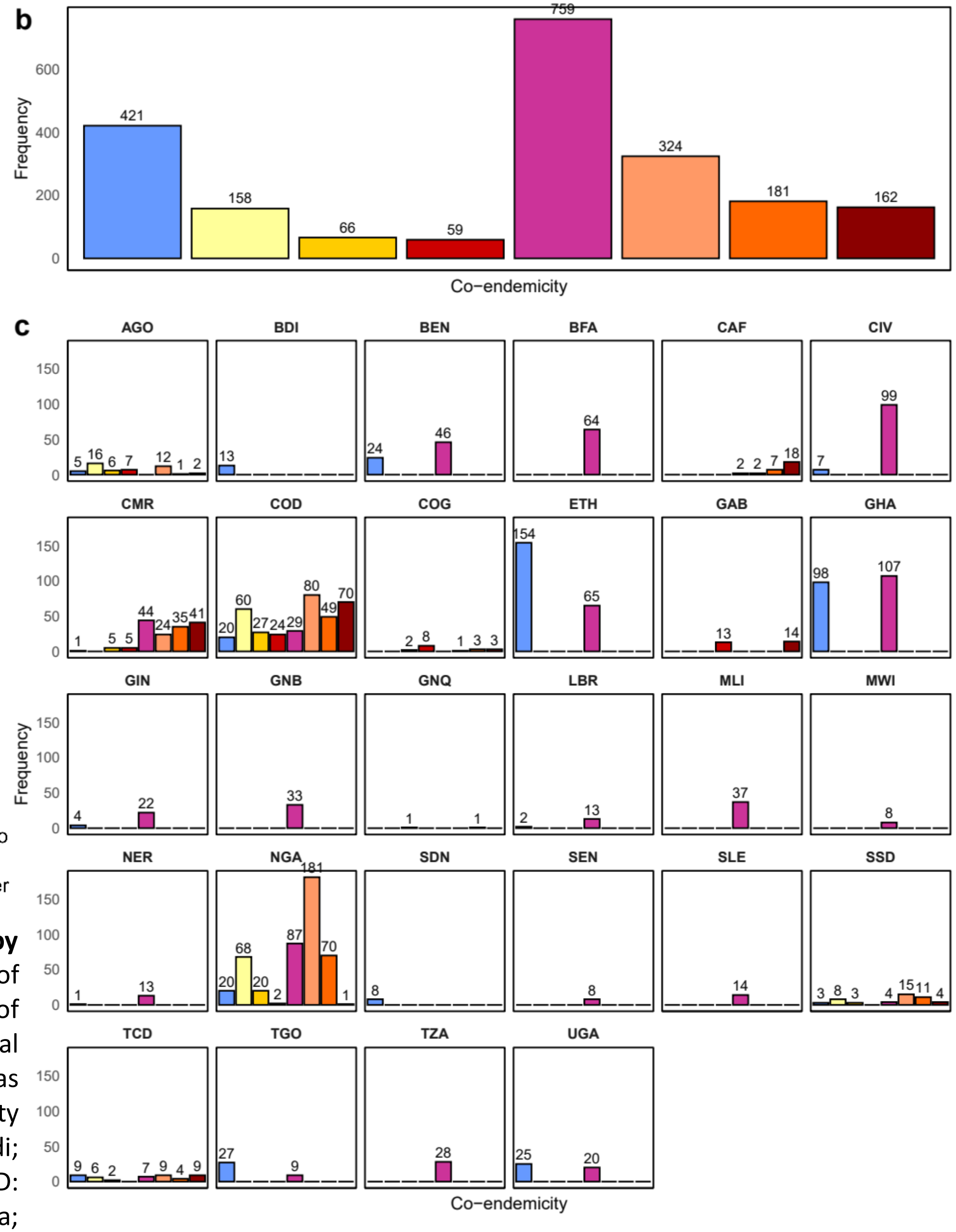

Fig. S2 Co-endemicity of onchocerciasis (oncho) with lymphatic filariasis (LF) and loiasis (loa) by implementation unit (IU). Classifications of co-endemicity status are presented: (**a**) across a map of endemic countries, (**b**) as a frequency distribution across all IUs, and (**c**) as a frequency distribution of IUs within each country. IUs are indicated by grey borders. Inset map shows Bioko Island (Equatorial Guinea) in (**a**). IUs in grey are either non-endemic or with no intervention history (classified as 'Unknown' or 'Not reported' under the "Endemicity" variable in ESPEN)[1]. Loiasis endemicity classifications in the ESPEN loiasis database[8] are according to RAPLOA[9,10]. AGO: Angola; BDI: Burundi; BEN: Benin; BFA: Burkina Faso; CAF: Central African Republic; CIV: Côte d'Ivoire; CMR: Cameroon; COD: Democratic Republic of the Congo; COG: Republic of Congo; ETH: Ethiopia; GAB: Gabon; GHA: Ghana; GIN: Guinea; GNB: Guinea Bissau; GNQ: Equatorial Guinea; LBR: Liberia; MLI: Mali; MWI: Malawi; NER: Niger; NGA: Nigeria; SDN: Sudan; SEN: Senegal; SLE: Sierra Leone; SSD: South Sudan; TCD: Chad; TGO: Togo; TZA: Tanzania; UGA: Uganda.

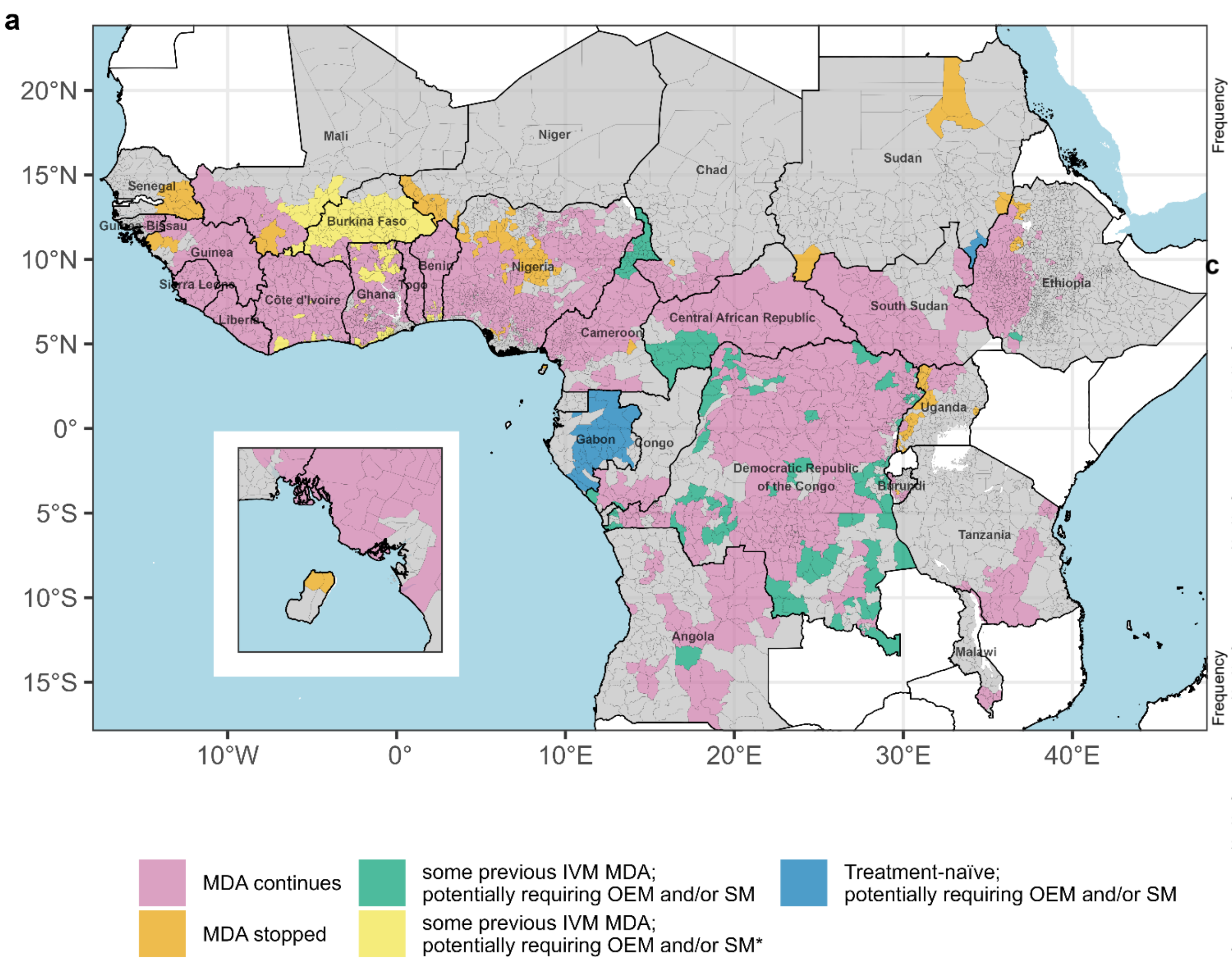

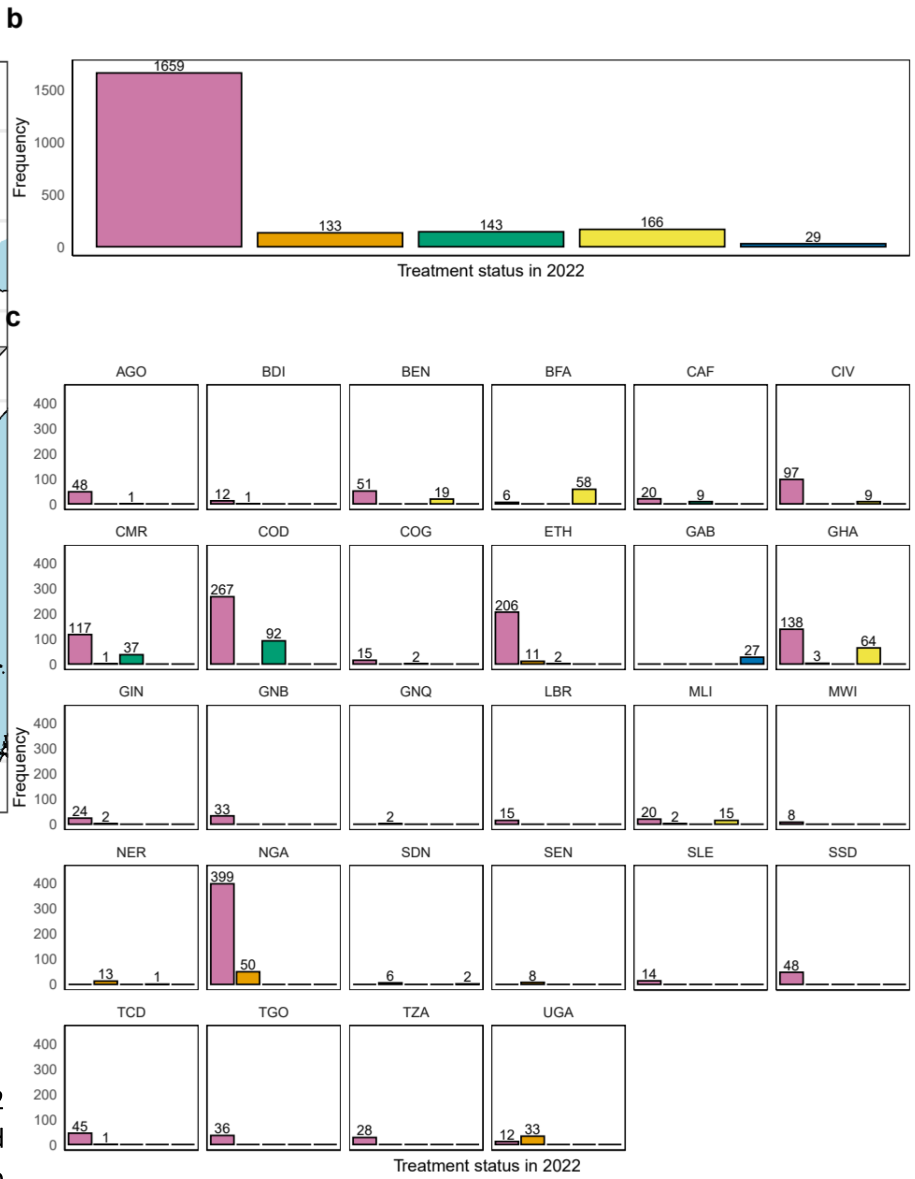

Fig. S3 Ivermectin mass drug administration (MDA) treatment status in 2022. Treatment status in 2022 is presented: (**a**) across a map of endemic countries, (**b**) as a frequency distribution across all IUs, and (**c**) as a frequency distribution of IUs within each country. IUs are indicated by grey borders. Inset map shows Bioko Island (Equatorial Guinea) in (**a**). IUs in grey are either non-endemic or with no intervention history (classified as 'Unknown' or 'Not reported' under the "Endemicity" variable in ESPEN)[1]. Country abbreviations are as in Fig. S2. OEM: Onchocerciasis Elimination Mapping[11]; SM: Suitability Mapping[12]; (*): indicates IUs in former OCP countries potentially requiring OEM/SM.

References

1. Expanded Special Project for the Elimination of Neglected Tropical Diseases (ESPEN) (2022). https://espen.afro.who.int/diseases/onchocerciasis (Accessed 13 February 2026).
2. O'Hanlon, S. J. *et al.* Model-based geostatistical mapping of the prevalence of *Onchocerca volvulus* in West Africa. *PLoS Negl Trop Dis* **10,** e0004328 (2016).
3. Zouré, H. G. M. *et al.* The geographic distribution of onchocerciasis in the 20 participating countries of the African Programme for Onchocerciasis Control: (2) pre-control endemicity levels and estimated number infected. *Parasit Vectors* **7,** 326 (2014).
4. Coffeng, L. E. *et al.* Onchocerciasis: the pre-control association between prevalence of palpable nodules and skin microfilariae. *PLoS Negl Trop Dis* **7,** e2168 (2013).
5. Coffeng, L. E. Onchocerciasis: the pre-control association between prevalence of palpable nodules and skin microfilariae - technical note and posterior draws for conversion equation (2024). https://zenodo.org/records/13969100 (Accessed 13 February 2026).
6. WHO/APOC. The WHO African Programme for Onchocerciasis Control final evaluation report. World Health Organization (2015). https://cdn.who.int/media/docs/default-source/documents/evaluation/evaluation-onchocerciasis-control.pdf?sfvrsn=f812abe5_2 (Accessed 13 February 2026).
7. Stolk, W. A., Borsboom, G., J. J. M. & Habbema, J. D. F. Trends in infection prevalence in the Special Intervention Zones of the former OCP and future expectations. Final report. 21 November 2011. Department of Public Health, Erasmus MC, University Medical Center Rotterdam, The Netherlands (2011).
8. Expanded Special Project for the Elimination of Neglected Tropical Diseases (ESPEN) (2021). https://espen.afro.who.int/diseases/loiasis (Accessed 13 February 2026).
9. Wanji, S. Rapid assessment procedures for loiasis: Report of a multi-centre study. UNDP/World Bank/WHO Special Programme for Research & Training in Tropical Diseases, TDR/IDE/RP/RAPL/01.1, Geneva (2001). https://fctc.who.int/resources/publications/i/item/rapid-assessment-procedures-for-loiasis-report-of-a-multi-centre-study (Accessed 13 February 2026).
10. Zouré, H. G. M. *et al*. The geographic distribution of *Loa loa* in Africa: results of large-scale implementation of the Rapid Assessment Procedure for Loiasis (RAPLOA). *PLoS Negl Trop Dis* **5,** e1210 (2011).
11. World Health Organization. Onchocerciasis elimination mapping: a handbook for national elimination programmes. Geneva: World Health Organization (2024). https://www.who.int/publications/i/item/9789240099227 (Accessed 13 February 2026).
12. Cromwell, E. A. *et al*. Predicting the environmental suitability for onchocerciasis in Africa as an aid to elimination planning. *PLoS Negl Trop Dis* **15,** e0008824 (2021).
